# Role of Human Mesenchymal Stem Cells in Regenerative Therapy

**DOI:** 10.3390/cells10010054

**Published:** 2020-12-31

**Authors:** Jayavardini Vasanthan, Narasimman Gurusamy, Sheeja Rajasingh, Vinoth Sigamani, Shivaani Kirankumar, Edwin L. Thomas, Johnson Rajasingh

**Affiliations:** 1 Department of Bioscience Research, University of Tennessee Health Science Center, Memphis, TN 38163, USA; jayavardini@gmail.com (J.V.); ngurusam@uthsc.edu (N.G.); srajasin@uthsc.edu (S.R.); vsigaman@uthsc.edu (V.S.); shivaani.kirankumar@gmail.com (S.K.); elthomas@uthsc.edu (E.L.T.); 2 Department of Genetic Engineering, SRM Institute of Science and Technology, Chennai 600036, India; 3 Department of Microbiology, Immunology and Biochemistry, University of Tennessee Health Science Center, Memphis, TN 38163, USA; 4 Department of Medicine, University of Tennessee Health Science Center, Memphis, TN 38163, USA

**Keywords:** mesenchymal stem cells, regenerative therapy, differentiation, tissue engineering

## Abstract

Mesenchymal stem cells (MSCs) are multipotent cells which can proliferate and replace dead cells in the body. MSCs also secrete immunomodulatory molecules, creating a regenerative microenvironment that has an excellent potential for tissue regeneration. MSCs can be easily isolated and grown in vitro for various applications. For the past two decades, MSCs have been used in research, and many assays and tests have been developed proving that MSCs are an excellent cell source for therapy. This review focusses on quality control parameters required for applications of MSCs including colony formation, surface markers, differentiation potentials, and telomere length. Further, the specific mechanisms of action of MSCs under various conditions such as trans-differentiation, cell fusion, mitochondrial transfer, and secretion of extracellular vesicles are discussed. This review aims to underline the applications and benefits of MSCs in regenerative medicine and tissue engineering.

## 1. Introduction

### 1.1. Regenerative Medicine—An Overview

Until recently, the study of tissue or organ regeneration was limited by religious and philosophical constraints. New developments and advancement in the fields of embryology and stem cells have led to the pursuit of regenerative medicine without constraints [1]. Tissue engineering and stem cell research are integral to regenerative medicine, the term introduced by Leland Kaiser in 1992 [2]. In regenerative medicine, cells are made to regenerate or replace the cells or tissues that are in a damaged and/or non-functional state [3]. This covers a combination of therapeutic approaches such as biocompatible materials, medical devices, artificial organs, and various cellular therapies.

**Figure 1:**
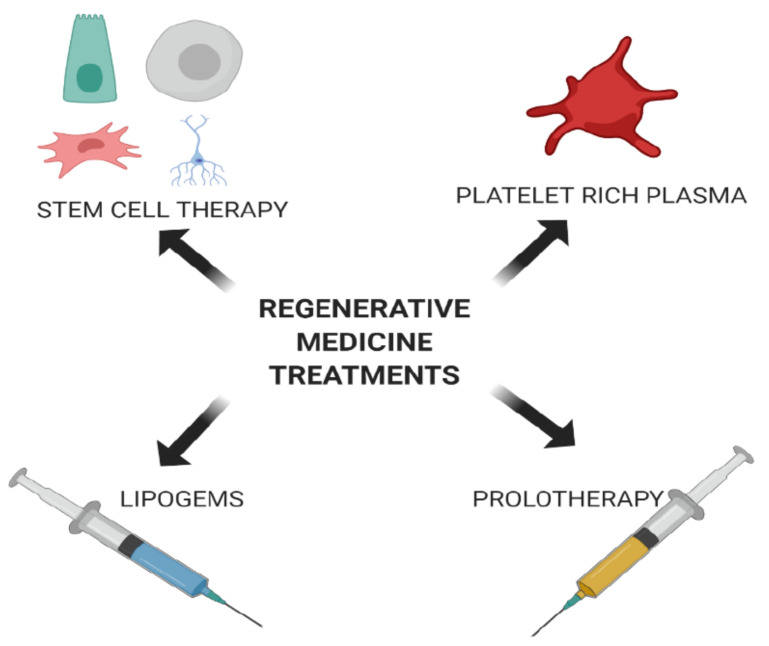
Various therapies employed in regenerative medicine. Currently, therapies utilizing various stem cells, platelet rich plasma, lipogems (adipose tissue), and prolotherapy (using an irritant such as dextrose) have been employed to regenerate or replace damaged cells or tissues.

Current treatment methods involving allogenic cells or other tissue components can pose complications to the patient including immunological rejection. However, autologous therapy using cells of the same individual avoids rejection and is a safe form of therapy. Autologous cell therapy is based on the patient’s needs and takes into account problems that may arise during treatment. Currently, four important regenerative approaches, stem cell therapy, platelet rich plasma (PRP) therapy, lipogems, and prolotherapy are available (Figure 1).

PRP therapy uses the patient’s own platelets to repair injuries and enhance healing [4]. Lipogems therapy uses micro-fractured adipose tissue, which contains heterogeneous populations of cells including Mesenchymal Stem Cells (MSCs). The adipose tissue is subjected to mild mechanical forces to obtain cells for treatment [5]. Prolotherapy uses natural substances, including stem cells from various sources and PRP to repair joint and tendon injuries [6]. In this review, we will focus on stem cell therapies, particularly those using MSCs, which are widely used to treat various diseases.

### 1.2. Stem Cells in Regenerative Medicine

In the late years of the twentieth century, studies defined the unique properties of stem cells [7]. Stem cells have the ability to self-renew, but also to differentiate in response to the environment in which they have been placed or directed towards. Researchers began to understand the working principles of differentiation of stem cells into various cell types through bone marrow transplantation studies [8]. Stem cells are classified as totipotent, pluripotent, multipotent, and unipotent based on their ability to differentiate into new cell line(s). The cells lose their stemness upon differentiation and maturation. Efforts are being made to maintain stem cells in the state in which they were derived or isolated by providing an appropriate environment that supports an undifferentiated state [9]. Controversy surrounded the use of pluripotent embryonic stem cells, in that these cells are isolated from blastocyst-stage embryos, requiring destruction of the embryo [10]. This led to studies of adult stem cells, the multipotent stem cells found in tissues and organs of every adult. These cells are responsible for maintaining the body’s functions by replacing cells that are dying and/or losing their tissue- or organ-specific function. An advantage of using adult stem cell is that the cells can be isolated or derived from the person in a diseased state or with cell/tissue loss. It is less controversial in that it is done with consent of the patient. Therefore, adult stem cell research has boomed and gained more attention [11,12]. Of the adult stem cells, hematopoietic stem cells (HSC) and mesenchymal stem cells (MSC) are the most widely used, mainly because they can be obtained from people in diseased states. The following sections focus on the behavior and characteristics required for applications of adult MSCs in regenerative medicine and tissue engineering.

### 1.3. Adult MSCs in Regenerative Medicine

MSCs have the potential for self-renewal and limited differentiation. They exist in many different tissues and organs such as adipose tissue, bone marrow, skin, fallopian tube, cord blood, liver and lungs [13]. MSCs were defined as stromal cells of the bone marrow and showed the properties of hematopoietic stem-like cells but were unable to differentiate into hematopoietic cells [14]. It has often been pointed out that studies of MSCs and their use in regenerative medicine face issues such as the difficulty of characterizing the cells and maintaining a homogenous culture [15]. However, current research has broadened the scope of techniques for working with MSCs, and these problems are now a relatively minor setback. MSCs have been found to be highly valuable for ongoing and future intensive research, including the phenomena of immunomodulation, angiogenesis, anti-apoptosis, anti-fibrotic activity, and chemo-attraction [16,17]. In addition, MSCs are able to support the growth and differentiation of other stem cells. Their capacity to secrete bioactive components is a major advantage in regenerative medicine [18,19]. Regenerative medicine takes into account these properties and has led to development of treatments for a wide range of human diseases, including diseases affecting the lungs, liver, heart, kidney, etc., [18]. Given these properties, it is evident that MSCs will hold a significant therapeutic role in medical and healthcare services and will lead many medical scientists to initiate clinical trials (Table 1).

## 2. Mechanism of Actions of Adult MSCs

### 2.1. Trans-Differentiation

Trans-differentiation is defined as a process by which a cell is made to differentiate from one cell type to another distinct lineage through genetic reprogramming [24]. MSCs have the ability to trans-differentiate into various germ layers such as ectoderm, mesoderm and endoderm. Bone marrow derived MSCs trans-differentiate under specific conditions into ectodermal derivatives such as neurons. These MSC-derived neurons were used to treat diseases associated with neurodegeneration by implanting them into a precise location [25,26]. Furthermore, MSCs were able to trans-differentiate into spindle-shaped Schwann cells, which were then used to treat neurodegenerative demyelination disorders and also to treat skin injuries [27,28]. These studies have proven that MSCs are able to trans-differentiate into cells belonging to the ectodermal germ layer. Moreover, MSCs have been shown to trans-differentiate under specific conditions into mesoderm germ layer cells such as adipocytes, osteocytes and chondrocytes. MSCs were able to trans-differentiate into osteoblasts and osteocytes that can be used for bone repair and regeneration [29]. Several studies have shown that MSCs can be trans-differentiated into adipocytes and chondrocytes [24,30,31]. MSCs also trans-differentiated into hepatocytes, which represents the endoderm lineage, and these cell types were used to treat liver diseases [32].

### 2.2. Cell Fusion

**Figure 2:**
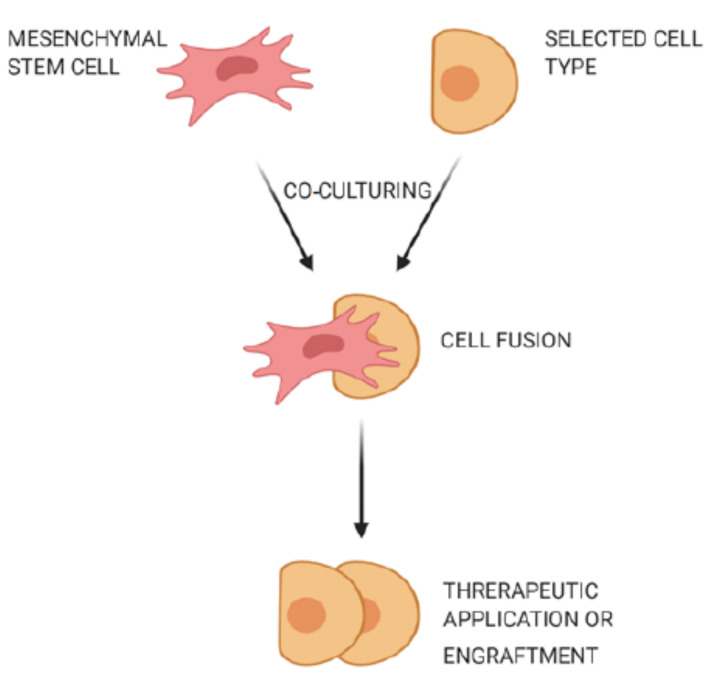
Fusion of mesenchymal stem cells with non-stem cells. Mesenchymal stem cells interact with neighboring cells to form a multicellular aggregate with improved characteristics, and these fused cells can be used for treatment of neurodegenerative or gastrointestinal disorders.

Cell fusion is defined as the process of one cell interacting with neighboring cells to form a multicellular aggregate with a common function (Figure 2). In this process, the genetic information is fused and expressed as molecular markers that show characteristic features of the fused cells [33]. Fusion of human MSCs with rodent cerebellar Purkinje cells was observed, and the fused cells were used to improve the therapy of neurodegenerative disorders or cerebellar related issues [34]. MSCs could also fuse with human gastrointestinal epithelial cells to form aggregates while expressing characteristic features of both cell types [35].

### 2.3. Mitochondrial Transfer

**Figure 3:**
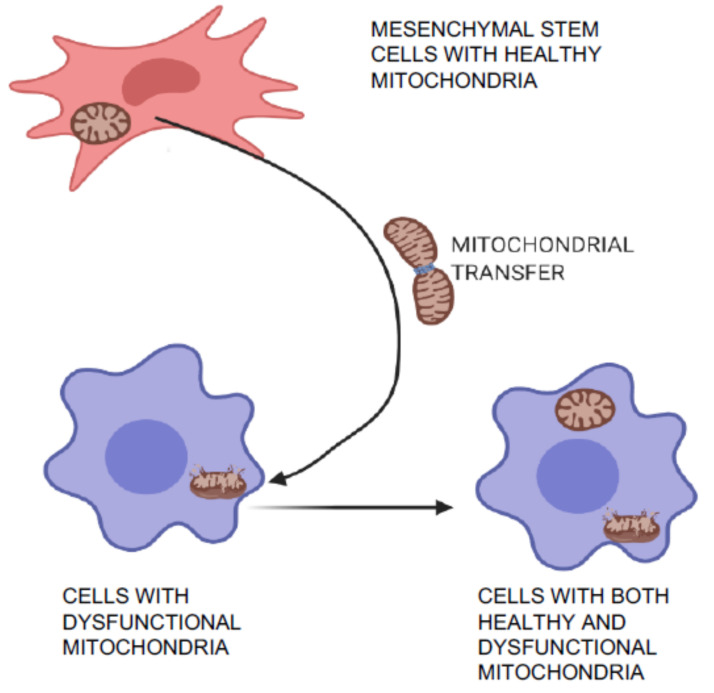
Mitochondrial transfer and repair of damaged cells. MSCs can transfer healthy mitochondria to the injured neighboring cells with dysfunctional mitochondria. This characteristic feature of MSCs help to regenerate several tissues including lung, heart, kidney, and brain.

Mitochondrial transfer is one of the unique phenomena of MSCs in which the cells can transfer mitochondria to neighboring injured cells, which are then restored (Figure 3) and can aid in tissue repair and regeneration [36,37]. Initially, transfer was proved by co-culture experiments when mitochondria from human MSCs were found in recipient cells that had been devoid of mitochondria [36]. Mitochondrial transfer was observed to occur through formation of intracellular nanotubes, gap junctions, cell fusion, microvesicles, and direct uptake of isolated mitochondria [38,39,40,41]. Mitochondrial transfer from MSCs plays a crucial role in regeneration of several tissues including lung, heart, kidney, and brain [42]. Many stress signals, such as the release of damaged mitochondria, mitochondrial DNA, and an increased level of reactive oxygen species induced the transfer of mitochondria from MSCs to recipient cells [41,43]. The expression of Miro1 protein in MSCs was shown to play an important role during tunnel tube formation for mitochondrial transfer to the injured cell under stress [44,45]. This feature of MSCs was found to repair cardiomyocytes during myocardial infarction [46,47], damaged corneal epithelium in the eye [48], renal tubular cells [49], brain-cortical cells [50], and lung cells [51].

### 2.4. Extracellular Vesicles or Microvesicles

Extracellular vesicles are released by many types of cells, including MSCs. The size of these vesicles ranges from 30–120 nm and they are called exosomes. The most important capabilities of these vesicles are to transport essential macromolecules like protein and genetic material to neighboring cells by endocytosis [52]. However, currently, these vesicles pose difficulties with extraction, quality, and reproducibility from differing cell types. MSC-derived extracellular vesicles or exosomes were utilized for cell-free therapy [53,54,55]. MSC-derived exosomes have been employed to overcome the transplant-associated problem of Graft vs. Host Disease (GvHD) [56]. Studies have shown that MSC-derived paracrine factors induce signaling, promote improved skin wound healing [57], and also mediate essential physiological functions [58]. Although some therapeutic benefits of MSCs are obtained through trans-differentiation and most of the benefits are mediated through paracrine mechanisms [59], it is worth noting that therapeutic effects of microvesicles and exosomes derived from MSCs differ depending on the tissue or organ in which they resided [60]. Thus, further studies are needed to compare exosomes derived from MSCs of differing origins.

## 3. Required Characteristics of MSCs for Their Application in Regenerative Medicine

### 3.1. Colony Formation

A colony forming unit (CFU) describes the ability of a single cell to multiply and form a colony of cells of its own kind. Colony formation is considered one of the primary functional characteristic features of MSCs and reveals the proliferation, differentiation, and survival of MSCs [61]. MSC colonies can be grown, fixed, stained and observed under a stereomicroscope for counting. Other phenotypic properties such as expression of cell surface molecules CD73, CD90 and CD105 help to identify, enrich and isolate colonies of MSCs [62,63,64].

### 3.2. Surface Phenotypes

Cell-surface markers are molecules that act as a fingerprint to identify the unique characteristics of a cell. The fingerprint consists of the specific molecules used for cell–cell interactions and recognition and provides quantitative phenotypic expression. Analyzing cell-surface markers is a well-known method to characterize MSCs. Cells adherent in standard conditions and which express CD105, CD73, and CD90 but not CD45, CD34, or CD14 or CD11b, CD79alpha or CD19, and HLA-DR surface markers are considered to be MSCs [65]. Although characterization of the surface phenotypic is used widely, it does pose ambiguity, depending on the cell source, method of isolation, and means of marker detection [66].

### 3.3. Plasticity and Differentiation Potential

Plasticity refers to the ability of stem cells to differentiate into many types of cell. It is an important characteristic of stem cells in regenerative therapies [67,68]. The surface markers and the differentiation potential are key requirements for characterization of MSCs [65]. In addition, the ability of MSCs to undergo trilineage differentiation (adipo-genic, chondrogenic and osteogenic) sets them apart from most other stem cells. Numerous studies have reported on the trilineage differentiation characteristics of MSCs. As a result, we now have a definite set of protocols that can be used to direct the differentiation pathway of MSCs for regenerative therapy [69].

### 3.4. Telomerase Activity

Telomeres are the end caps of chromosomes which shorten with replication and division. Telomere shortening leads to cell senescence. Telomerase is the enzyme responsible for replication of the telomeric region, and increased telomerase activity is directly associated with a longer lifespan for a cell. In MSCs, the telomerase activity is increased, providing an added advantage for use in regenerative therapies that pose age-related hindrances [70]. Telomerase activity and telomere lengths can be used as a quality control measure to select MSCs for therapy [71].

## 4. Application of MSCs in Tissue Engineering

Regenerative medicine is an exciting field of research with amazing outcomes. Several tissue engineering technologies have been used in regenerative medicine, and currently there are three powerful technologies that have been broadly applied: bioprinting, use of scaffolds, and organoid technologies.

### 4.1. Bioprinting Technology

**Figure 4:**
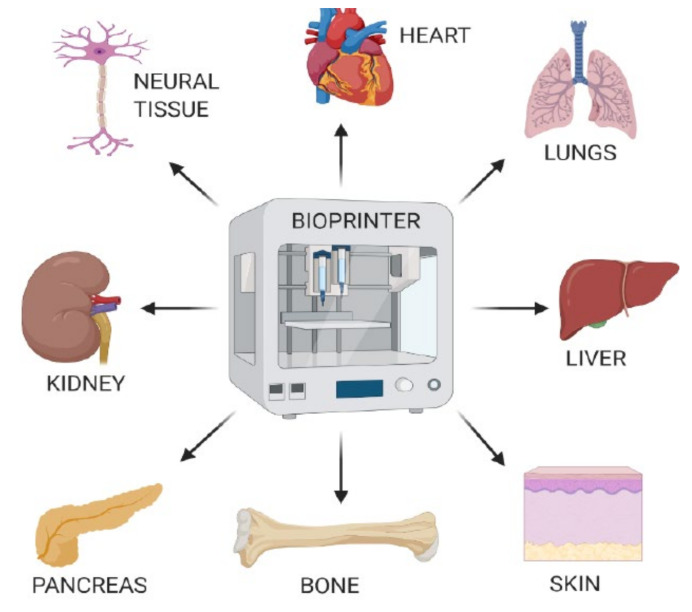
Design and generation of various tissues or organs using 3D-Bioprinting Technology. Bioprinting technology facilitates the fabrication of bioconstructs utilizing MSCs and other biomaterials in a hierarchical manner to produce various tissues or organs, which can be used for regenerative therapy.

Bioprinting is a state-of the-art technology for building tissue and organ structures similar to their original counterparts. It allows us to fabricate bioconstructs with cells and biomaterials in a hierarchical manner following a layer-by-layer process exactly in predefined locations [72]. Bioprinting using human MSCs has been successfully applied to generate bone tissue. Hydroxyapatite nanoparticles were found to effectively promote osteogenic differentiation of MSCs [73]. Another study showed that bioprinting MSCs along with growth factors such as bone morphogenetic protein 2 and transforming growth factor β1 helped to engineer an anisotropic biomimetic fibrocartilage microenvironment [74]. Various tissues and organs can be designed and generated using this 3D-Bioprinting Technology [75] (Figure 4).

### 4.2. Scaffolds

Scaffolds play a crucial role in tissue engineering strategies because they provide an architecturally fit environment for the cells to grow on. A scaffold should be able to accommodate the cells of interest and provide growth factors to help in maintenance, proliferation, and differentiation of the cells. The scaffold should exhibit mechanical properties suitable for reconstructing the damaged tissue. Scaffolds used in regenerative therapy should be biodegradable in the human body after the scaffold delivers the cells of interest to the desired area [76]. Initially, scaffolds were manufactured from organic acids, but later studies found that the acids were toxic to the cells and damaged the microenvironment [77]. To overcome this problem, researchers invented and began use of a new technology called additive manufacturing. This technology consists of selective addition of biomaterials to form a complex three-dimensional structure.

**Figure 5:**
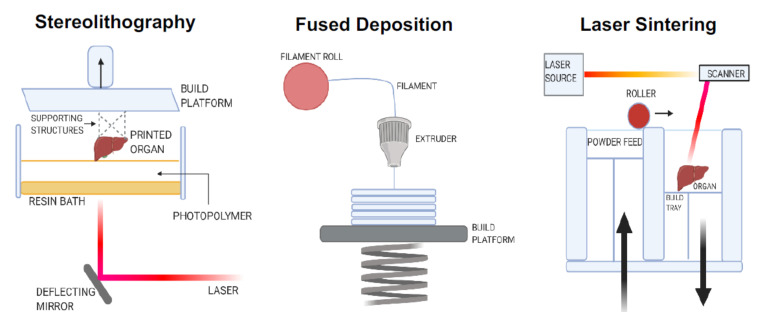
Types of manufacturing process in organoid technology. Organoid technology is the in vitro development of a three-dimensional structure to mimic the original architecture of tissues and organs, using various stem cells. Three different categories of additive manufacturing processes employed in organoid technology are stereolithography, fused deposition manufacturing, and selective laser sintering.

There are three different categories of additive manufacturing processes: stereolithography, fused deposition manufacturing, and selective laser sintering. Stereolithography is a solid freeform technique, which allows the fabrication of parts by a computer aided design [78]. Using the stereolithographic technique, the scaffold resolution can be controlled up to several µm, and the scaffold can be customized to match the targeted tissue for replacement [79]. Fused deposition manufacturing is a computer-controlled protrusion and deposition process, which is employed to create very complex honeycomb-like structured scaffolds [80]. Selective laser sintering has the advantage of rapid prototyping. This method rapidly constructs complex scaffolds with predesigned macro- and micro-structures [81] (Figure 5).

MSCs proliferate at much higher rates in a scaffold than on plastic dishes. Their survival and differentiation potentials are also higher when grown in scaffolds. Gingival MSCs cultured on a poly (lactic acid) scaffold had a much higher potential to become differentiated into neural cell types than when cultured without a scaffold and can be used to treat diseases associated with neurodegeneration [82]. Biodegradable nanofibrous scaffolds loaded with MSCs showed good potential for chondrogenesis, which provides a promising path for cartilage tissue engineering [83].

### 4.3. Organoid Technology

Organoid technology is the in vitro development of a three-dimensional structure, along with stem cells, to mimic the original architecture of tissues and organs, forming a reasonably close replica of a natural structure [84]. Thus, organoid technology represents a useful research tool in bridging the gap between two-dimensional cell cultures and in vivo animal models. This technology is also a biologically-relevant cell-based method for screening drugs, which is a major challenge in drug discovery. Normal adult kidney specimens can be propagated in vitro into organoid three-dimensional structures composed of both differentiated and undifferentiated cells expressing nephron-specific markers [85]. The organoid models using the individual’s own somatic cells, either MSCs or induced pluripotent stem cells (iPSCs), more closely represent the organ microenvironments, cell-to-cell interactions, and cell signaling that occur in vivo.

The research using organoid technology opened doors for many discoveries. For instance, a group of researchers were able to use MSCs to generate an organoid that has been stimulated to produce an analogue of human erythropoietin, which in turn helps battle anemia in rats. This organoid was a near-perfect substitute that could be transplanted into a host with minimal or no complications [86]. Therefore, modified MSCs can be used to produce organoids with multiple therapeutic applications. Intestinal organoids were used as tissue surrogates for pharmacological and toxicological studies [87]. Adult stem cells from healthy and diseased humans can be used to generate three-dimensional organoid structures that can be used to study organ development and also disease-modelling [88]. These organoids can be used for applications including cancer therapy [88,89] and also have potential for organ transplantation [90,91,92]. The applications of MSCs in the field of tissue engineering are principally due to the multifactorial characteristics of MSCs.

## 5. Induced Pluripotent Stem Cell-Derived MSCs (iMSCs)

**Figure 6:**
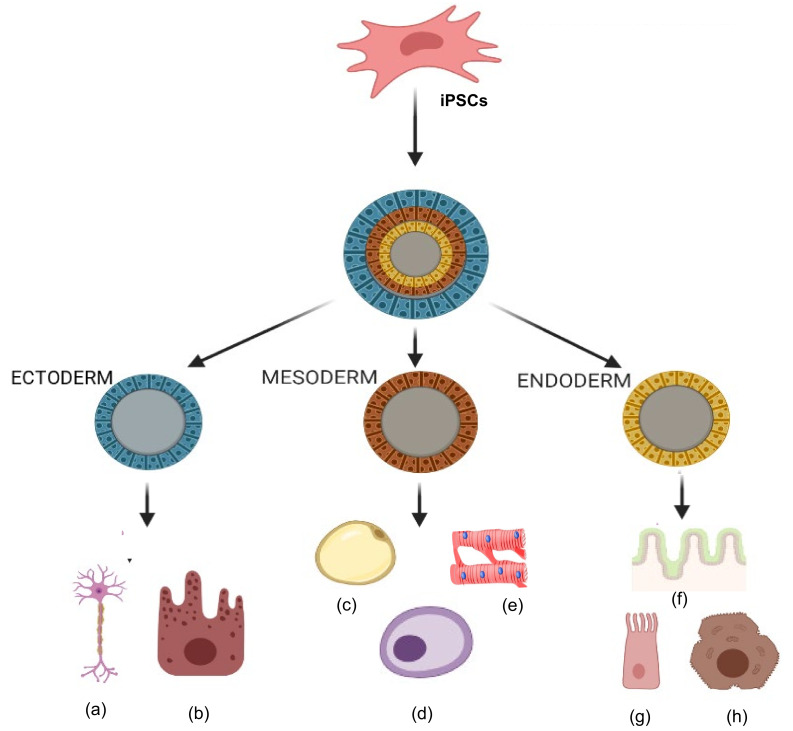
Tri-lineage differentiation capabilities of induced pluripotent stem cells (iPSCs). Ability of iPSCs to differentiate into three germ layers (ectoderm, mesoderm and endoderm) in types of cells such as (**a**) neurons, (**b**) epithelial cells, (**c**) adipocytes, (**d**) osteocytes, (**e**) cardiomyocytes, (**f**) gut epithelium, (**g**) lung cells, and (**h**) hepatocytes.

Organ or tissue repair with cell therapy has emerged as a promising treatment alternative in patients with degenerative diseases. Researchers are currently developing a variety of therapies with stem cells obtained from many different sources and which can provide trophic and paracrine support or even replace dying cells [93]. A fundamental goal of regenerative medicine is to repair the failing organ by replenishing functional cells. A variety of autologous and allogeneic cell types have been tested for repair of diseases in humans, including cardiac diseases, and have shown a wide range of results, from significant improvement to no improvement [94,95,96]. MSCs isolated from adult organs hold promise, but scalability and senescence are major issues. During insult to any organ, cells are lost or become dysfunctional, and the post-insult milieu can have a negative impact on the health of autologous MSCs and their therapeutic capabilities. Thus, manipulation of autologous cells into primitive iPSCs and then further differentiation into specific required cell types is an attractive approach in stem cell transplantation therapy (Figure 6). Studies have shown that iMSCs promote healing in inflammatory bowel disease [97] and limb ischemia [98]. Moreover, iMSCs promote angiogenesis in ischemia models [99] and prevent allergic airway inflammation [100].

## 6. Challenges Associated with MSCs for Regenerative Medicine and Tissue Engineering

MSCs are of great interest in therapeutic research due to their multifunctional characteristics. MSCs exhibit powerful properties such as self-renewal, in vivo differentiation, and immunomodulation, which make them an excellent cell source for treatment of deadly disorders. Despite the many advantages of MSCs in regenerative therapy, there is a need to understand the challenges faced and find ways to overcome them. Major challenges of MSCs are associated with their isolation, processing, and safety requirements. The source of the MSCs plays a vital role in expression of their therapeutic properties. In addition, the age, sex, health conditions, surgical conditions, etc. of cell donors play an important role in successfully isolating MSCs [101]. The characterization of MSCs also poses challenges. After isolation and selection of MSCs, one must ensure that the MSCs are therapeutically active, pure, untransformed, and functional. Throughout the processes from isolation to therapeutic application, microbiological safety and other cell culture safety measures must be followed, including the good laboratory practice/good manufacturing practice (GLP/GMP) regulations. [102,103].

To use MSCs in cell delivery treatments, one must face the challenge of ensuring that the microenvironment of the host meets the requirements for the specific type of cell. It was found that the host environment led to the development of cell types other than those needed for therapy [104]. It was also found that an inflammatory environment (a specific composition of active immune cells along with mediators such as TLR ligands) was a conditional requirement for MSCs to modulate immune reactions for therapy. It can be difficult to create such an environment [105]. MSCs pose a few problems like immuno-rejection or tumorigenesis when transplanted to human or animal hosts. In addition, MSCs have some adverse effects in transplanted organs and may induce blood clotting [106]. The side effects of MSCs depend on the source of the cells as well as on their route of administration [18]. As we move on to the steps to be taken for the effective use of MSCs, we must find ways to combat these problems. In the future, applications of MSCs can be enhanced through genetic modifications, chemical engineering, and preconditioning technologies. Another method will be utilization of iMSCs in place of the usual tissue-derived MSCs [98,100].

## 7. Conclusions

The broad applications of MSCs are mainly due to their regenerative potentials as well as their immunomodulatory characteristics. Clinical studies have demonstrated therapy with adult MSCs from various sources has the ability to repair injured tissues. Recently, the discovery of iMSCs from controlled differentiation of iPSCs to obtain a homogeneous population represents a promising alternative to adult MSCs for regenerative therapy. In this review, we have addressed both the advantages as well as the challenges associated with applications of MSCs. Future studies should focus on manipulating and reconstituting MSCs and iMSCs to enhance migration, adhesion, and paracrine trophic factor secretion with emphasis on relevance to healthcare.

## Figures and Tables

**Table 1:** Some of the clinical trials using mesenchymal stem cells and or other stem cells.

S. No	Title of the Clinical Trial	Year and Identifier	Current Status	Particulars
1	Amnion bilayer and stem cell combination therapy on thin endometrium infertile patients	2020-NCT04676269	Early Phase-I	Amnion epithelial cells for infertile patients. www.clinicaltrials.gov.
2.	A phase I/IIa, open label, dose-escalating clinical study to evaluate the therapeutic effects of astrocytes derived from human embryonic stem cells, in patients with Amyotrophic Lateral Sclerosis (ALS)	2018-NCT03482050	Phase-II	Intrathecal mode of injected one time at varied doses to the ALS patients. www.clinicaltrials.gov.
3	Safety and efficacy of Bone Marrow-derived autologous stem cells for the treatment of Duchenne muscular dystrophy	2017-NCT03067831	Phase II	To check the muscle strength www.clinicaltrials.gov.
4.	Hematopoietic stem cells and mesenchymal stem cells (MSCs) for severe Aplastic Anaemia	2014	Completed	Active positive response was observed with some limitations [20].
5.	Long term follow up phase I/II, open, multi-center, prospective study using human embryonic stem cell derived retinal pigmented epithelial for patients with age-related macular degeneration (AMD).	2015-NCT02463344	Completed	No signs of hyperproliferation, tumorigenicity, ectopic tissue formation, or apparent rejection after 4 months [21]. www.clinicaltrials.gov.
6.	Graft versus host disease (GVDH) treated using bone marrow derived MSCs	2013	Phase III	No clear adverse effects associated with MSC infusion were observed [22].
7.	Safety and efficacy Phase I study of Umbilical Cord/Placenta-(UC) derived MSCs to treat Ankylosing Spondylitis (AS).	2011-NCT01420432	Completed	UC-MSC along with disease-modifying anti-rheumatic drugs served as anti-inflammatory and immunomodulatory agents [23]. www.clinicaltrials.gov.

## Data Availability

Not applicable.
